# Mental Health Predictors of Dynamic Balance in Individuals With Chronic Ankle Instability

**DOI:** 10.1002/jfa2.70152

**Published:** 2026-03-30

**Authors:** Abbis Jaffri

**Affiliations:** ^1^ Department of Physical Therapy Creighton University Omaha Nebraska USA

**Keywords:** balance, kinesiophobia, lower extremity, rehabilitation

## Abstract

**Background:**

Chronic ankle instability (CAI) is linked to impaired dynamic balance and psychological deficits, potentially hindering optimal rehabilitation outcomes. However, the impact of psychological deficits on dynamic balance has not been explored before. Therefore, the aim of this study is to determine whether mental health factors predict dynamic balance performance in individuals with CAI using the Star Excursion Balance Test (SEBT).

**Methods:**

This is a cross‐sectional observational study performed in a university setting. Forty individuals with CAI (17 men, 23 women) participated in this study. Assessments included psychological profiles (kinesiophobia, self‐reported mental health, self‐efficacy, global mental health, and functional status via FAAM‐Sport). Dynamic balance was evaluated using the SEBT composite and directional scores.

**Results:**

Multiple linear regression analyses indicated that the SEBT composite score was significantly predicted by self‐efficacy, global mental health and TSK‐11, explaining 40% of the variance (*F*‐value = 7.82, *p* < 0.01). Further directional analysis revealed that posterior‐medial reach was best predicted by self‐efficacy, global mental health and TSK‐11 (*F*‐value = 9.05, *p* < 0.01; 44% variance), while posterior‐lateral reach was similarly predicted by self‐efficacy, and global mental health (*F*‐value = 6.92, *P* = 0.003; 28% variance). Anterior reach performance was most strongly associated with self‐efficacy and TSK‐11 (*F*‐value = 10.26, *p* < 0.01; 36% variance).

**Conclusions:**

The findings underscore the importance of psychological factors as key predictors of dynamic balance in CAI. Incorporating mental health assessments into physical rehabilitation may enhance treatment outcomes for individuals with CAI.

**Abbreviations**
CAI: Chronic ankle instability
DPA: Disablement in physically active
FAAM: Foot and ankle ability measure
IdFAI: Identification of functional ankle instability
LAS: Lateral ankle sprain
MCS: Mental component summary
PCS: Physical component summary
SF‐12: The short form health survey
TSK‐11: 11‐item Tampa scale of kinesiophobia
VAS: Visual analog scale

## 1 Introduction

Lateral ankle sprains (LASs) are highly prevalent injuries in both active and general populations [1]. Nearly 70% of individuals who experience a LAS develop chronic ankle instability (CAI) within a year, characterized by persistent pain, weakness and limited function [2, 3]. In the United States (U.S.), approximately 2 million ankle sprains occur annually, with more than 3 million hospital visits reported due to ankle sprains in 2010 [4]. This shows that a large number of the US population is at risk of developing CAI and its associated co‐morbidities, which can impact daily life [2].

Individuals with CAI often experience impaired dynamic balance. Postural control deficits are repeatedly reported in individuals with CAI [5, 6, 7] and are considered the most important sensorimotor impairment. Sensorimotor deficits in ankle instability include impairments in joint position sense, peroneal muscle reaction time, and balance [2]. Additionally, sensorimotor impairments also include differences in static and dynamic balance between healthy and CAI groups [8]. Star Excursion Balance Test (SEBT) was primarily used to evaluate dynamic balance in individuals with CAI, including various other assessment tools such as Dynamic Leap and Balance Test [6, 9].

SEBT performance measures balance by having individuals reach as far as possible in several directions while balancing on one leg. Other factors can also impact SEBT performance such as range of motion (ROM), flexibility, and strength [10, 11]. Previous studies have shown that reach distance on the SEBT is affected by ROM deficits at the hip, knee, and ankle [12, 13]. However, it is unclear whether psychological factors influence dynamic balance in individuals with CAI. Previous work has shown that verbal encouragement can improve SEBT reach distances in individuals with CAI [14], suggesting a potential influence of psychological factors on SEBT performance. Considering that impaired dynamic balance may increase the risk of recurrent ankle sprains and associated co‐morbidity in individuals with CAI, further research is needed to explore psychological predictors of dynamic balance as measured by the SEBT.

Several studies have reported elevated levels of kinesiophobia and fear‐avoidance beliefs in people with chronic musculoskeletal injuries [15, 16, 17], indicating a high prevalence of mental health concerns in this population. Furthermore, individuals with CAI reported more self‐perceived disablement, poor health‐related quality of life, and fear of reinjury in comparison to a healthy population [17]. Additionally, several psychological factors, such as perceived ankle instability and kinesiophobia have been included within the sensory‐perceptual impairments of the current CAI model [2]. While psychological impairments have been shown to affect functional performance, however, the influence of mental health on dynamic balance in individuals with CAI remains unclear. Two established theoretical frameworks provide a foundation for understanding how psychological factors may mechanistically influence dynamic balance in CAI. The Fear‐Avoidance Model posits that following musculoskeletal injury, fear of reinjury and pain catastrophizing drive kinesiophobia, which engages supraspinal inhibitory mechanisms that attenuate voluntary motor output and constrain movement amplitude during demanding tasks such as the SEBT [15, 16, 17, 18]. Complementarily, Bandura's Self‐Efficacy Theory proposes that individuals with lower perceived capability may adopt protective movement strategies which for individuals with CAI can be hypothesized by reduced range of motion and conservative reach distances that compromise balance performance independent of physical impairments [14, 19]. Together, these frameworks suggest that psychological states may actively modulate the neuromuscular and attentional processes underpinning dynamic postural control in individuals with CAI.

Therefore, our study aims to determine the mental health predictors of dynamic balance in individuals with CAI. Understanding the impact of mental health on dynamic balance will provide insight on the role of psychological impairments in functional outcomes. We hypothesize that higher levels of negative psychological beliefs such as kinesiophobia (TSK‐11 scores) and lower levels of positive psychological beliefs such as self‐efficacy would predict poorer performance (shorter reach distances) in all SEBT directions (anterior, posteromedial, and posterolateral), whereas higher levels of positive psychological beliefs such as self‐efficacy and global mental health would predict better performance (greater reach distances) across all SEBT directions.

## 2 Methods

This cross‐sectional study was approved by the Institutional Review Board of Creighton University.

### 2.1 Participants

Forty individuals with CAI participated in this study. Table 1 presents the demographics of the participants. Inclusion criteria were as follows: (1) history of at least one ankle sprain (initial sprain at least 1 year prior to participation), (2) score of < 85% on the Foot and Ankle Ability Measure Sport Scale (FAAM‐Sport), and (3) score of > 10 on the Identification of Functional Ankle Instability (IdFAI) questionnaire [1, 20, 21, 22]. The FAAM‐Sport survey assesses sport related physical function, and the IdFAI questionnaire assesses an individual's self‐perceived ankle instability status. Participants were excluded if they had sustained an ankle sprain within the past six weeks or had any balance or neurological disorders or an active clinically diagnosed psychological or psychiatric condition. Written informed consent prior to participation, along with demographic data (age, sex, injury history) and anthropometric measurements (height, weight, limb length, injured limb) were obtained [1, 20, 21, 22].

**TABLE 1:** Patient demographics (mean ± standard deviation).

Outcomes	Overall (*n* = 40)
Sex	23 Female, 17 male
Age (years)	23.92 ± 4.11
Height (cm)	173.64 ± 8.95
Weight (kg)	76.66 ± 13.06
FAAM‐sport	18.52 ± 3.89
IdFAI	18.75 ± 7.60
TSK‐11	21.00 ± 5.54
SF‐12 mental	51.11 ± 9.07
SF‐12 physical	45.05 ± 8.64
Self‐efficacy	66.38 ± 17.81
Global health mental	15.53 ± 2.95
DPA	23.15 ± 11.16

Abbreviations: DPA = Disablement in Physical Activity, FAAM‐sport = Foot and ankle ability measure‐sport, IdFAI = Identification of functional ankle instability, SF‐12 Mental = 12‐Item Short Form Survey‐Mental Component, SF‐12 Physical = 12‐Item Short Form Survey‐Physical Component, TSK‐11 = 11‐item Tampa scale of kinesiophobia.

*A formal* a priori power analysis was not conducted. Post hoc, we used G*Power (v3.1; F tests; Linear multiple regression: fixed model, deviation from zero) based on observed values from the final models. With *N* = 40, *α* = 0.05, and 2–3 predictors, the corresponding effect sizes were *f* ^2^ = 0.39–0.79 (*R* ^2^ = 0.28–0.44), yielding achieved power between 0.81 and 0.96, exceeding the conventional 0.80 threshold. Sensitivity analysis further indicated that, with *N* = 40 and *α* = 0.05, the minimum detectable effects for 80% power were *f* ^2^ = 0.28 for 2 predictors and *f* ^2^ = 0.34 for 3 predictors; our observed *R* ^2^ values (0.28–0.44) exceed these thresholds. The final models contained 2–3 predictors, yielding subject‐to‐predictor ratios of 13:1 to 20:1, which are within commonly cited guidelines of 10–15 participants per predictor. Furthermore, the sample size of this study is consistent with prior observational studies examining psychological and sensorimotor predictors of functional outcomes in individuals with CAI [9, 23]. Although nine candidate predictors were initially considered, best subsets regression with Mallows' Cp criterion reduced the final models to 2–3 predictors.

### 2.2 Procedure

A baseline of clinical assessment including the self‐reported psychological and functional outcomes were assessed for each participant. After baseline testing, participants performed dynamic balance assessments using the Star Excursion Balance Test. Data collection was conducted between May 2023 and January 2024.

### 2.3 Mental Health

Psychological outcomes associated with CAI were evaluated using a series of validated questionnaires administered prior to functional testing. The Global Health scale (v.1.0/1.1) assessed physical and mental health through 10 items rated on a 5‐point Likert scale (1 = poor to 5 = excellent), with raw scores converted to *T*‐scores (mean = 50, SD = 10) using standardized scoring algorithms. *T*‐scores range from 20–80, where higher scores indicate better health status. In this study, only the Mental sub‐score was used as a measure for mental well‐being. The 11‐item Tampa Scale of Kinesiophobia (TSK‐11) [24], a short‐form survey, measured fear of movement using a 4‐point Likert scale (1 = strongly disagree to 4 = strongly agree) for each item, yielding total scores ranging from 11–44, where higher scores indicate greater kinesiophobia. The short‐form SF‐12 Health Survey examined the impact of general health on daily life through two sub‐scales: the Physical Component Summary (PCS) for physical health and the Mental Component Summary (MCS) for mental health, both calculated using weighted scoring algorithms to produce norm‐based scores (mean = 50, SD = 10), with scores below 50 indicating below‐average health status [25]. The Disablement in the Physically Active (DPA) [24] survey assessed health‐related quality of life in active populations through 16 items rated on a 5‐point scale (1 = no problem to 5 = severe), producing scores from 0–64 after transformation, with higher scores reflecting greater disablement. Finally, a modified 17‐item self‐efficacy scale [26], utilizing a visual analog scale (VAS) ranging from 0% to 100%, evaluated participants' perceived ability to perform various functional tasks. The reliability for 17‐item self‐efficacy scale has previously been reported to be high (ICC_2,1_ = 0.79) in individuals with CAI. Each item is rated on a 100 mm VAS, with anchors at “no confidence at all” (0) and “completely confident” (100). The total score is calculated as the mean of all 17 items, providing a score range of 0–100. Higher scores indicate greater self‐efficacy for managing condition‐related challenges.

### 2.4 Dynamic Postural Control

Dynamic balance was assessed using the SEBT in three directions: anterior (Ant), posteromedial (PM), and posterolateral (PL). The SEBT is a reliable test in detecting reach distance differences between individuals with CAI and non‐injured individuals [9, 27]. To complete this task for each direction, participants were instructed to be completely barefoot while balancing on the limb with their affected ankle, and reach as far as possible with the non‐affected limb while keeping their hands on their hips. We repeated trials if any weight was placed on the reaching limb, hands were removed from the hips, the participant lost balance, or the toe did not come in contact with the floor. For the Ant direction, the toes were aligned with the zero mark. For the PM and PL directions, the heel was aligned with the zero mark. Reach was measured in centimeters as the distance of touch from the zero mark. Three trials were conducted, and an average reach measurement was taken in each direction. The average reach distances were divided by leg length and multiplied by 100 to normalize the SEBT scores. Leg length was measured from the anterior superior iliac spine to the superior aspect of the medial malleolus. The reach distances were normalized to account for leg length variations, as SEBT performance has been demonstrated to correlate with lower extremity length differences.

### 2.5 Data Analysis

Statistical analysis was conducted using Minitab (Minitab LLC, State College, PA, USA). The analysis followed a systematic stepwise approach: First, skewness, kurtosis, and Shapiro‐Wilk tests confirmed normal distribution of all dependent variables (SEBT directions and composite scores). Second, Pearson product‐moment correlations were calculated between all explanatory variables (DPA, 17‐item self‐efficacy scale, global mental health scale, global physical health, SF‐12 mental and physical scales, TSK‐11, FAAM‐sport, and IdFAI) and dependent variables to identify potential predictors; variables with moderate correlations (*r* ≥ 0.25) with any SEBT direction were retained as candidates. Third, multicollinearity was assessed by examining Pearson correlations among explanatory variables and calculating variance inflation factors (VIF), retaining only variables with VIF values close to 1. Fourth, best subsets regression was performed to evaluate all possible combinations of the retained predictors, followed by application of Mallows' Cp criterion to identify the optimal model for each outcome, balancing model fit with parsimony. Fifth, four multiple linear regression models were constructed using the predictors identified through this process: one for SEBT composite score and one for each direction (anterior, posteromedial, posterolateral). Finally, all regression assumptions (linearity, independence, normality, and homoscedasticity) were verified for each final model through residual analysis and appropriate diagnostic tests (attached as Supporting Information S1).

## 3 Results

This research involved 40 participants (17 men and 23 women). Table 1 provides details on individual demographic characteristics along with means and standard deviations for the variables used in this study.

Best subsets regression with Mallows' Cp identified a three‐predictor model (self‐efficacy, global mental health, and TSK‐11) as optimal for SEBT composite scores (*R* ^2^ = 0.40) (Table 2). Within this model, self‐efficacy and global mental health positively predicted the composite scores while TSK‐11 negatively predicted the composite scores. Self‐efficacy, global mental health and TSK‐11 collectively explained 40% of the variance (overall model: *F*‐value = 7.82, *p* < 0.01).

**TABLE 2:** Multivariable linear regression for dependent variable SEBT composite score.

Predictor	*B*	SE [*B*]	*p* value	VIF	R‐sq
Constant	46.73	9.92	0.000		0.40
Self‐efficacy	0.21	0.059	0.001	1.13	
Global health mental	0.55	0.37	0.15	1.23	
TSK‐11	−0.177	0.209	0.40	1.36	

Abbreviations: B = Coefficient, SE [*B*] = Standard error of the coefficient, VIF = Variance Inflation Factor.

Similarly, in the PL direction, self‐efficacy and global mental health emerged as the most significant subset of predictors, each positively contributing to PL reach performance based on the VIF analysis (Table 3). Together, self‐efficacy and global mental health accounted for 28% of the variance in PL reach performance (overall model: *F*‐value = 6.92, *P* = 0.003).

**TABLE 3:** Multivariable linear regression for dependent variable SEBT‐PL reach.

Predictor	*B*	SE [*B*]	*p* value	VIF	R‐sq
Constant	29.8	10.1	0.006		0.28
Self‐efficacy	0.25	0.09	0.01	1.03	
Global health mental	1.24	0.57	0.04	1.03	

Abbreviations: B = Coefficient, SE [*B*] = Standard error of the coefficient, VIF = Variance Inflation Factor.

For the PM reach, self‐efficacy, TSK‐11 and global mental health were identified as the key subset of predictors based on the VIF analysis (Table 4). Self‐efficacy and global mental health positively predicted the PM reach while TSK‐11 negatively predicted the PM reach. Self‐efficacy, TSK‐11, and global mental health explained 44% of the variance in PM reach performance (overall model: *F*‐value = 9.05, *p* < 0.01).

**TABLE 4:** Multivariable linear regression for dependent variable SEBT‐PM reach.

Predictor	*B*	SE [*B*]	*p* value	VIF	R‐sq
Constant	44.1	10.7	0.000		0.44
Self‐efficacy	0.26	0.06	0.002	1.13	
TSK‐11	−0.12	0.23	0.62	1.36	
Global health mental	1.01	0.400	0.016	1.23	

Abbreviations: B = Coefficient, SE [*B*] = Standard error of the coefficient, VIF = Variance Inflation Factor.

Finally, for the ANT reach, self‐efficacy and TSK‐11 were the most influential subset of predictors based on the VIF analysis, with self‐efficacy positively and TSK‐11 negatively predicting the ANT reach performance (Table 5). Self‐efficacy and TSK‐11 accounted for 36% of the variance in ANT reach performance (overall model: *F*‐value = 10.26, *p* < 0.01).

**TABLE 5:** Multivariable linear regression for dependent variable SEBT‐Ant reach.

Predictor	*B*	SE [*B*]	*p* value	VIF	R‐sq
Constant	36.84	3.60	0.000		0.36
Self‐efficacy	0.15	0.04	0.000	1.15	
TSK‐11	−0.21	0.14	0.142	1.15	

Abbreviations: B = Coefficient, SE [*B*] = Standard error of the coefficient, VIF = Variance Inflation Factor.

## 4 Discussion

Findings from this study indicate that the influence of psychological variables on SEBT performance was primarily driven by self‐efficacy, which showed consistent predictive value across reach directions. Other psychological measures exhibited limited or direction‐dependent effects, highlighting self‐efficacy as the most robust psychological correlate of balance performance on the SEBT in this CAI population. These results underscore the importance of psychological factors in SEBT performance for this population. Previous research has linked physical impairments, such as deficits in ankle dorsiflexion ROM [10, 11], to poor dynamic balance. However, the most novel contribution of this study is identifying psychological predictors of dynamic balance in individuals with CAI, an area not previously explored. Furthermore, recent evidence suggests that extrinsic motivational cues, such as verbal encouragement (VE), can significantly enhance reach distances‐particularly in the anterior and posteromedial directions [28]. The VE may have helped these individuals overcome supraspinal inhibitions, which are processed in the cerebral cortex and limbic system. This potentially breaks the psychological barriers that develop after injury, hinting at more robust yet holistic treatment strategies in physical rehabilitation. These strategies may include integrating psychological components into physical therapy for optimal functional outcomes [29]. Although, this is the first study to delineate such relationships in individuals with CAI, previous evidence has shown that psychological or mental health deficits, such as increased anxiety, cause instability in postural control in other clinical groups [30, 31]. Additionally, anxiety increases and efficacy decreases during more challenging balance tasks [30, 31], such as the SEBT, when compared to a static balance test [32]. Ohno et al. [30] showed that the effects of anxiety were diminished in an eyes closed balance, suggesting anxiety has a greater effect during tasks that involve visual processing.

While it's been shown that greater levels of perceived ankle instability are associated with decreased reported ankle function [23], our findings revealed that perceived ankle instability is not a strong predictor of dynamic balance. The data from Sutmiller et al. [23] indicate a disconnect between self‐reported perceptions of ankle function and performance on objective functional tests: on average, individuals with CAI rated their ankle function lower than what their test performance would suggest. In our sample, self‐efficacy was positively associated with balance performance, such that higher self‐efficacy related to better SEBT performance. Taken together, these findings suggest that psychological factors, particularly self‐efficacy, may help explain why self‐reports sometimes diverge from performance‐based assessments and point to self‐efficacy as a modifiable target to potentially improve functional outcomes.

Novel treatment approaches have been developed that attempt to address this gap in rehabilitative care. One of these treatment approaches is psychologically informed physical rehabilitation (PIPR), which incorporates psychological training such as pain management, cognitive restructuring, and goal achievement into physical rehabilitation programs [33]. Increasing evidence has been found in support of PIPR programs for improving psychological and functional outcomes in patients with various orthopedic issues [34, 35]. PIPR for patients with low back pain has been shown to be effective in reducing disability and pain and increasing health and function [36, 37]. It is possible that PIPR treatment for individuals with CAI may provide a solution for the mental health impairments that can exist even when physical function has been improved upon following an ankle sprain or other injury. Future research should investigate the effectiveness of PIPR treatment approaches for individuals with CAI.

This study has several limitations that should be considered when interpreting the findings. First, the dynamic balance was assessed solely using the SEBT, providing a narrow measure of functional performance. There is a need of new balance performance tasks that can purely assess the proprioceptive deficits without getting effected by the psychological impairments [32]. Second, the sample size of 40 participants, while demonstrating adequate post‐hoc statistical power, may limit the generalizability of the findings, and replication in larger samples is warranted. Third, the study sample consisted predominantly of young university students, which may limit the generalizability of findings to other populations such as professional athletes, recreational sport participants, or older adults with CAI, who may present with different psychological profiles and functional demands. Future studies should aim to recruit a larger sample size, look at the effects of mental health on other physical function parameters, and compare these results with dynamic balance measured using different tests.

### 4.1 Clinical Implications

We believe our research will encourage clinicians, particularly those specializing in musculoskeletal rehabilitation, to adopt a holistic, whole‐person approach when treating or designing rehabilitation programs for individuals with CAI. Persistent ankle instability and giving away can contribute to mental health challenges while also affecting self‐efficacy which augment poorer postural control. The chronicity of symptoms may create a detrimental cycle that adversely affects both the mental and physical well‐being of those with CAI. Patients have expressed concerns about not regaining normal function post‐injury, despite repeated assurances from clinicians [38]. We propose that similar anxieties are present in individuals with CAI, resulting in a decline in their overall mental and physical health. As a result, individuals recovering from an ankle sprain are typically cleared to return to sport or daily activities after physical impairments are improved. However, we currently see high rates of reinjury in the CAI population [1]. The results of this study suggest that even after completing rehabilitation, individuals may still experience mental health impairments that affect their balance, increasing their susceptibility to falls and further injury. Current rehabilitation practices often overlook psychological factors such as self‐efficacy and mental health. Our findings suggest that incorporating psychological assessments and interventions into CAI rehabilitation could enhance functional outcomes. Specifically, addressing self‐efficacy may help individuals achieve better balance performance, supporting more comprehensive return‐to‐activity decisions that account for both physical and psychological readiness. Therefore, psychological impairments in individuals with CAI should be regularly assessed and addressed as part of impairment‐based rehabilitation programs which currently only focus on motor behavior and arthromechanical impairments [2]. Similar recommendations are echoed in the recent clinical practice guidelines (CPG's) for the management of CAI where the authors of CPG's recommend the use of psychologically informed techniques, such as motivational interviewing, to maximize patients' self‐efficacy and to address uncomplicated psychological correlates and mediators of injury adjustment and recovery in order to maximize the effects of treatment in a positive manner for individuals with a LAS and CAI [39].

## 5 Conclusion

This study identified global mental health, self‐efficacy and TSK‐11 as significant predictors of dynamic balance performance in individuals with CAI. These findings highlight the critical role of psychological factors in dynamic balance and reinforce the need for a comprehensive approach that addresses both physical and mental health impairments. To effectively improve dynamic balance deficits and reduce recurrent ankle injuries, clinicians should integrate psychological assessments and interventions into CAI rehabilitation programs.

## Author Contributions

**Abbis Jaffri:** conceptualization, data curation, formal analysis, investigation, methodology, project administration, validation, visualization, writing – original draft, writing – review and editing.

## Funding

The author has nothing to report.

## Ethics Statement

The study was approved by the Institutional Review Board of Creighton University.

## Consent

The author has nothing to report.

## Conflicts of Interest

The author declares no conflicts of interest.

## Supporting information


Supporting Information S1

## Data Availability

Data and materials can be available upon request.
