# Supplementary material for: Mental Health Predictors of Dynamic Balance in Individuals With Chronic Ankle Instability
Source: J Foot Ankle Res. 2026 Mar 30;19(2):e70152. doi: 10.1002/jfa2.70152 (PMC13581079; doi:10.1002/jfa2.70152)
Supplement: Supplementary file 1 [file JFA2-19-e70152-s001.docx]

**Supplemental Material:** Residual Plots for Regression Assumption Verification

Introduction to Residual Analysis

Residual plots are essential diagnostic tools used to verify that the assumptions underlying linear regression models are satisfied. This supplemental material presents residual diagnostic plots for all regression models examining the relationship between psychological factors and Star Excursion Balance Test (SEBT) performance in individuals with chronic ankle instability.

Description of Residual Plots and Their Interpretation

1. Residuals vs. Fitted Values Plot Purpose: Evaluates linearity and homoscedasticity (constant variance) Interpretation:

- Points should be randomly scattered around zero
- No patterns, curves, or funnel shapes should be present

2. Normal Q-Q Plot Purpose: Assesses normality of residuals Interpretation:

- Points should follow the diagonal reference line
- Minor deviations at extremes are acceptable

3. Scale-Location Plot Purpose: Checks for homoscedasticity Interpretation:

- Points should scatter randomly with horizontal trend
- Consistent spread across fitted values

4. Residuals vs. Leverage Plot Purpose: Identifies influential observations and outliers Interpretation:

- Points should show no extreme leverage with large residuals

**Model 1: SEBT Composite Score**

**Assumption Verification:**

- Linearity: Residuals vs. fitted values plot shows random scatter around zero with no discernible pattern - SATISFIED
- Homoscedasticity: Scale-location plot demonstrates consistent variance across fitted values - SATISFIED
- Normality: Q-Q plot indicates residuals follow normal distribution with points aligning to reference line - SATISFIED
- No influential outliers: Residuals vs. leverage plot shows all observations within acceptable thresholds - SATISFIED

Model 2: Posterolateral (PL) Reach Direction

**Assumption Verification:**

- Linearity: Residuals display random distribution around zero line without systematic patterns - SATISFIED
- Homoscedasticity: Variance remains constant across range of fitted values - SATISFIED
- Normality: Q-Q plot confirms approximate normality of residuals - SATISFIED
- No influential outliers: No observations exceed critical leverage values - SATISFIED

**Model 3: Posteromedial (PM) Reach Direction**

**Assumption Verification:**

- Linearity: Random residual scatter confirms linear relationship between predictors and outcome - SATISFIED
- Homoscedasticity: Equal variance maintained across all levels of fitted values - SATISFIED
- Normality: Residuals follow normal distribution as shown in Q-Q plot - SATISFIED
- No influential outliers: All data points fall within acceptable influence thresholds - SATISFIED

**Model 4: Anterior (ANT) Reach Direction**

**Assumption Verification:**

- Linearity: No non-linear patterns present in residuals vs. fitted values plot - SATISFIED
- Homoscedasticity: Spread-location plot confirms homogeneous variance - SATISFIED
- Normality: Normal Q-Q plot demonstrates approximately normal distribution of residuals - SATISFIED
- No influential outliers: Leverage values remain within acceptable limits for all observations - SATISFIED

**Technical Notes**

Statistical analyses were conducted using Minitab (Version 20, Minitab LLC, State College, PA, USA). Diagnostic plots were generated using standard regression diagnostic procedures. No data transformations were required as all variables met distributional assumptions in their original scale.
